# A prospective observational study of the impact of an electronic questionnaire (ePAQ-PO) on the duration of nurse-led pre-operative assessment and patient satisfaction

**DOI:** 10.1371/journal.pone.0205439

**Published:** 2018-10-19

**Authors:** Sarah K. Taylor, John C. Andrzejowski, Matthew D. Wiles, Sarah Bland, Georgina L. Jones, Stephen C. Radley

**Affiliations:** 1 University of Sheffield, Sheffield, United Kingdom; 2 Department of Anaesthesia, Sheffield Teaching Hospitals NHS Foundation Trust, Sheffield, United Kingdom; 3 Pre-operative Assessment, Sheffield Teaching Hospitals NHS Foundation Trust, Sheffield, United Kingdom; 4 Leeds Beckett University, Leeds, United Kingdom; 5 Department of Obstetrics and Gynaecology, Sheffield Teaching Hospitals NHS Foundation Trust, Sheffield, United Kingdom; Jinling Clinical Medical College of Nanjing Medical University, CHINA

## Abstract

**Objective:**

Standard pre-operative assessment at our institution involves a comprehensive history and examination by a nurse practitioner. An electronic pre-operative assessment questionnaire, ePAQ-PO® (ePAQ, Sheffield, UK) has previously been developed and validated. This study aimed to determine the impact of ePAQ-PO on nurse consultation times and patient satisfaction in low-risk patients.

**Methods:**

The duration of pre-operative assessment consultation was recorded for American Society of Anesthesiology physical classification 1 and 2 patients undergoing pre-operative assessment by an electronic questionnaire (ePAQ-PO group) and standard face-to-face assessment by a nurse practitioner (standard group). Patients were also asked to complete an eight-item satisfaction questionnaire. Eighty-six patients were included (43 in each group).

**Results:**

After adjusting for the duration of physical examination, median (IQR [min-max]) consultation time was longer in the standard compared to the ePAQ-PO group (25 (18–33 [10–49]) min vs. 12 (8–17 [4–45]) min, respectively; p <0.001). Response rate for the satisfaction questionnaire was 93%. There was no significant difference in patient satisfaction scores (38/39 in standard group vs. 39/41 in ePAQ-PO group were fully satisfied with their pre-operative assessment; p = 0.494).

**Conclusion:**

Pre-operative assessment using ePAQ-PO is associated with a significant reduction of over 50% in the duration of the assessment without impacting on patient satisfaction.

## Introduction

Nurse-led pre-operative assessment is now commonplace in elective surgical pathways in many hospitals [1,2]. The role of the pre-operative assessment physician is multi-faceted with several key components: optimisation of patient health to try and reduce peri-operative complications [3,4]; management of co-existing medical conditions in accordance with latest guidance [5,6]; provision of patient education as part of peri-operative care bundles [7,8]; ensuring that appropriate, patient-centred investigations are undertaken [9,10]; and provision of information relevant to consent for surgery [11]. It is recognised that well trained nursing staff can play an ever-increasing role in pre-operative assessment providing they are well trained [12,13]. Attendance at pre-operative assessment is used opportunistically for health-screening purposes [1] and medication reviews [14]. These increased demands, paired with limited resources and an aging population, mean that new and innovative solutions need to be found to ensure patients receive timely and appropriate pre-operative assessment.

In response to this need, we have developed and validated an electronic pre-operative assessment questionnaire, ePAQ-PO® (ePAQ, Sheffield, UK) [15]. This is completed by the patient at a desktop computer or an online program, and using a series of structured questions, takes a full medical history. It employs skipping rules to make completion of the form more rapid. The ePAQ-PO output includes a summary that can be annotated by clinicians, as well as an estimate of the patient’s ASA physical classification. American College of Cardiology/American Heart Association guidelines suggest that pre-operative cardiac evaluation is tailored to the circumstances and nature of the surgery [16]. The value of airway examination is also of limited value in predicting peri-operative problems [17]. Physical examination and airway evaluation is not carried out in our department in the cohort of patients undergoing ePAQ-PO evaluation. Although ePAQ-PO has been shown to be as accurate as clinicians at predicting ASA score, and is reliable and easy for patients to complete [15], hitherto evidence has been lacking regarding its impact on care pathways.

The primary aims of this study were to compare the duration of nurse consultation in low-risk patients undergoing pre-operative assessment using ePAQ-PO with those undergoing standard pre-operative assessment, and to assess patient satisfaction with the two types of pre-operative assessment. Both these aims were achieved in the study.

## Methods

This was a prospective observational cohort study conducted from November 2016 to February 2017. The study was registered with the service improvement department (Mr P Griffiths, Director) at Sheffield Teaching Hospitals and ethical approval (Ref 001260) was obtained from the University of Sheffield ethics committee on 31^st^ October 2016 (Ms Laura Williams, administrator). As ePAQ-PO is already in routine use within our institution, individual patient consent was not deemed necessary by either committee.

Any patient attending pre-operative assessment who was aged ≥18 years and ASA physical classification 1–2 was eligible for inclusion. We did not study patients who could not speak fluent English or were unable to use a computer. Recruitment was carried out on three days per week over a four-month period.

**Fig 1:**
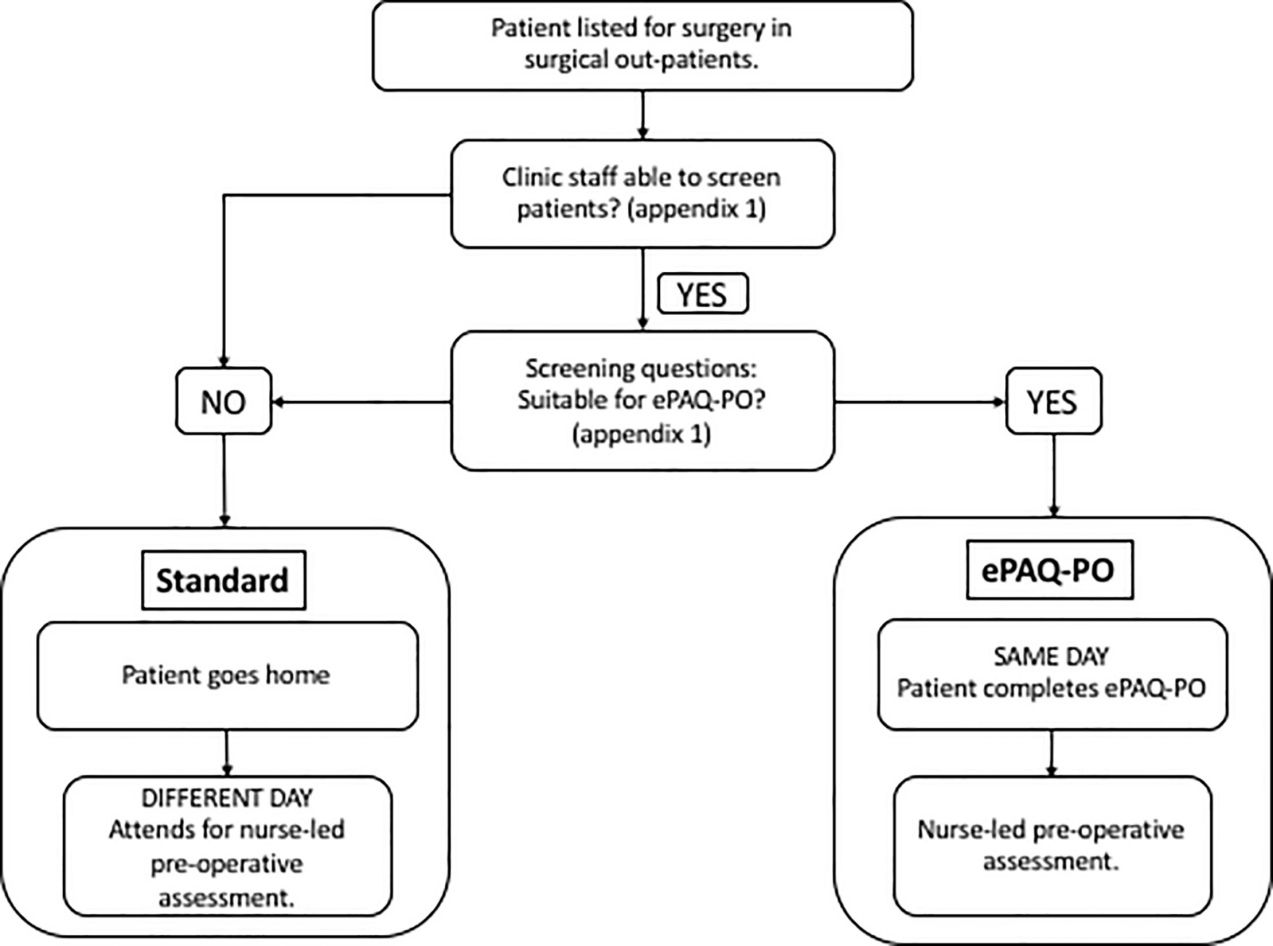
Patient pre-operative assessment pathways in use at Sheffield teaching hospital NHS foundation trust.

Patients in our institution undergo two types of pre-op assessment (Fig 1). Patients using ePAQ-PO are assessed on the same day as being listed for surgery, whereas standard care patients attend the hospital for their pre-operative assessment on a subsequent day following their surgical out-patient appointment. An anaesthetist is present at all times in the clinic to oversee the work of the nurses and practitioners and for advice or further consultation in more complicated patients. All the nurse practitioners employed have undergone a postgraduate course and been awarded a university diploma in pre-operative assessment. Our previous work has shown them to be as accurate as consultant anaesthetists and ePAQ-PO in allocation of ASA status [15]. The nurses follow the NG45 guidelines published by the National Institute for Health and Care Excellence (NICE) on which investigations to order in all patients [18]. Until the advent of ePAQ-PO, standard pre-operative assessment consisted of an appointment with a nurse practitioner, where a proforma is used to take a structured history, and a physical examination performed (standard group). This consists of auscultation of the heart and lungs and examination of the airway for estimation of ease of tracheal intubation. Alternatively, patients who are likely to be ASA physical class 1 or 2, may now be eligible to have electronic pre-operative assessment; ePAQ-PO. At the time of this study, a screening questionnaire (Appendix 1) was used by the clerical staff in the surgical outpatient clinic (ie not in the preassessment clinic) to determine if a patient could be offered this alternative ‘one-stop’ pre-assessment pathway. This short questionnaire was designed to be a simple screening tool to facilitate the smooth running of the ePAQ-PO pathway. Its main purpose was to prevent ASA > 2 patients from entering the ePAQ-PO pathway after it had been initially set up. (If an occasional ASA 3 or 4 patient attended, they were encouraged to fill in ePAQ-PO before seeing a nurse practitioner). Patients who agree, then complete ePAQ-PO on a computer in the pre-operative assessment clinic and subsequently have a consultation with a staff nurse to review the ePAQ-PO output; these patients do not undergo a physical examination (ePAQ-PO group) after departmental review of its intrinsic value in relatively young and healthy patients [16,17]. Standard patients were recruited from any patients who did not fulfil the screening criteria as described above, as well as those referred from surgical outpatient clinics that were unable to screen patients (Fig 1).

At the end of the consultation, both groups receive identical information and advice about their forthcoming admission and surgery. All patients have routine observations, microbiology swabs and any other relevant investigations carried out separately to their consultations as part of their pre-operative assessment visit.

The duration of pre-operative assessment consultation with the nurse (from time of patient entry into clinic room to the time of patient exit) for standard and ePAQ-PO groups was recorded by the same independent assessor. This duration included the time taken to write in the patients’ notes and/or annotate the computer record. Since ePAQ-PO patients are not physically examined, the duration of the physical examination in 20 patients in the standard group was also recorded. This allowed a constant to be produced which was used to adjust the consultation times for the standard group. In order to assess the patient satisfaction of each pre-operative assessment pathway, after each timed consultation the patient was offered an eight-item questionnaire which used a five-point Likert scale assessing agreement with statements relating to their experience.

The study was powered on the primary outcome measure of duration of nurse-led consultation. Previous work undertaken in our pre-operative assessment clinic found that the mean (SD) duration of pre-operative consultation was 47 (12) min [19]. In order to demonstrate a 10-minute decrease in consultation time with ePAQ-PO with 95% power and two-sided significance of < 0.05, we calculated that 37 patients would be required in each group. To account for an estimated 15% drop-out rate, we therefore aimed to recruit 43 patients in each group. We have previously shown that the duration of pre-operative assessment is affected by patient age and ASA physical class [19]. The formula for standard assessment derived in this paper was:

## Assessment time (min) = 48 + 0.17 (age) +/- (constant for given ASA Status)

Thus, a five-year difference might be expected to make a difference of 1 minute to median consultation time between groups. ASA status was assigned by the nurse after each consultation. To ensure ASA class and age matching (within 5 years) between the two groups, recruitment continued until we achieved balanced cohorts. To limit selection bias, recruitment took place on different days of the week on a rotational basis to ensure patients from a wide range of surgical specialties were included in the study and patients who were unable to speak English to a suitable level or who stated that they could not use a computer mouse, were not recruited to the standard group. Patients were assessed by one of only four nurse practitioners and two staff nurses in the standard and ePAQ-PO groups respectively.

Distribution of data were assessed by inspection of histograms and Kolmogorov-Smirnoff tests. Independent t-tests and Mann Whitney U tests were performed on age and consultation duration data, with Fisher’s Exact Tests used to assess patient satisfaction scores between the two groups.

## Results

**Fig 2:**
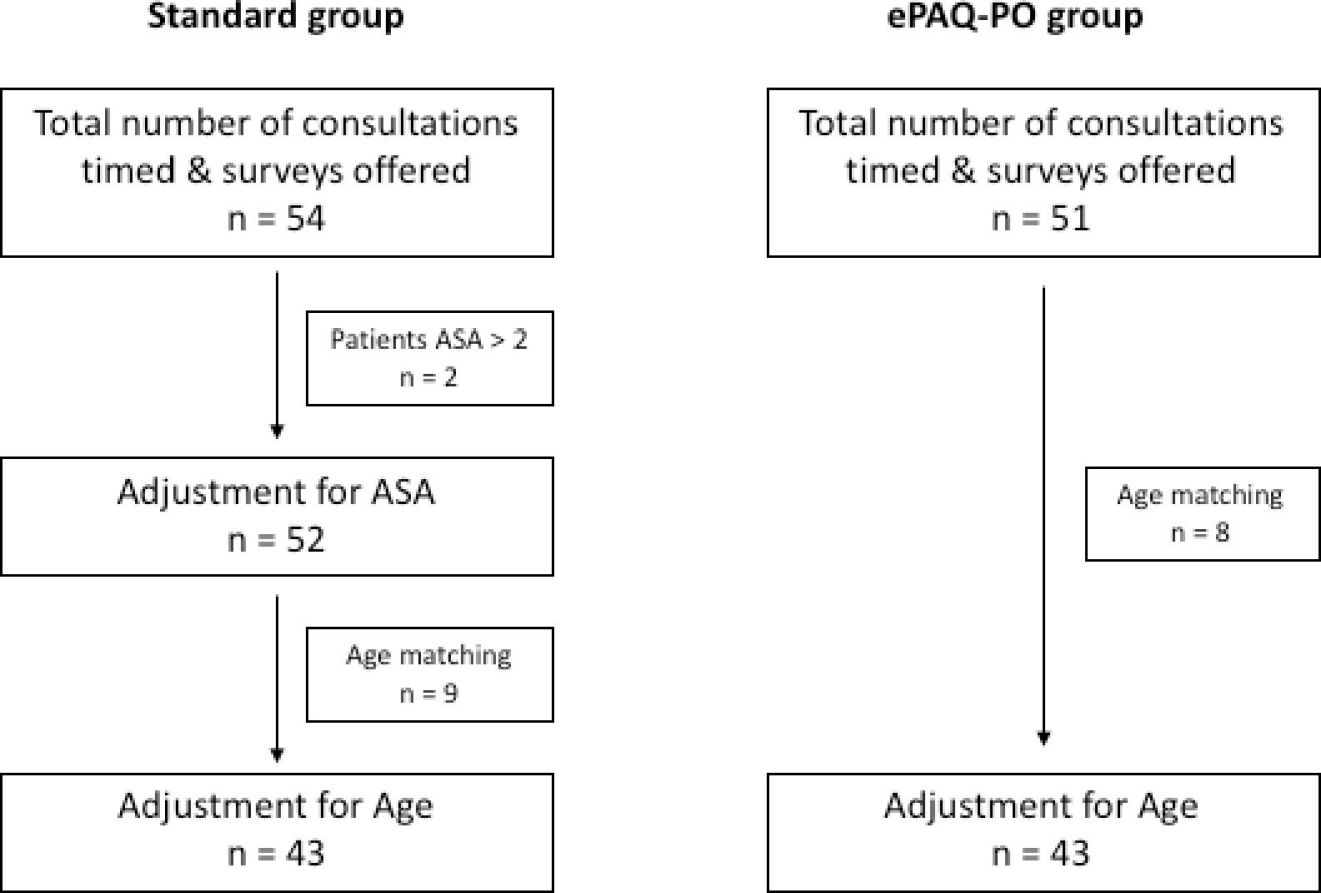
Recruitment flowchart and matching of groups.

In order to achieve age and ASA matched cohorts, 54 and 51 patients were recruited to the standard and ePAQ-PO groups respectively (Fig 2), with 43 patients analysed in each of the two groups.

There was no difference in the mean age or number of female patients in each group. Median consultation time was significantly longer in the standard compared to ePAQ-PO group, 29 min vs 12 min, respectively; p <0.001). Physical examination added a median of 4 min to the duration of the standard group consultations. When the data were adjusted to account for the physical examination, the statistically significant difference persisted in the median consultation duration in standard vs ePAQ group, 25 min vs 12 min, respectively; p <0.001. (Table 1)

**Table 1:** Demographic data and results for standard vs ePAQ-PO groups. Times are median (IQR [min-max]) values. Adjusted consultation time = measured consultation time—examination time.

	Standard	ePAQ-PO	p value
Number of patients	43	43	
Males	18	21	
Females	25	22	
ASA 1	5	17	
ASA 2	38	26	
Mean (SD) age in years	45 (14)	42 (12)	0.18
Consultation time (mins)	29 (22–37 [14–53])	12 (8–17 [4–45])	<0.001
Examination time (mins)	4 (3–5 [2–10])	0	<0.001
Adjusted consultation time (mins)	25 (18–33 [10–49])	12 (8–17 [4–45])	<0.001

Eighty patients (response rate 93%) completed the satisfaction questionnaire. Over 90% of patients in each group agreed or strongly agreed that they were fully satisfied with their pre-operative assessment, and 95% in each group reported communication with their pre-operative assessment nurse was good. There was no statistically significant difference in patient satisfaction scores for any of the items (Table 2). There were no ‘on the day’ cancellations or peri-operative complications for any of the patients in the study.

**Table 2:** Grouped satisfaction data for patients undergoing nurse-led pre-operative assessment with face-to-face consultation (standard group) or with the addition of an electronic questionnaire (ePAQ-PO group). Values shown are the number of patients who agreed or strongly agreed with the statements / total number of patients in each group who answered the question.

	Standard group	ePAQ-PO group	P value
I know which medications to stop before my operation.	33 / 34	25 / 27	1.000
My pre-operative assessment took too long	3 / 38	9 / 41	0.062
Communication with the pre-operative assessment team was good	38 / 39	39 / 41	1.000
My pre-operative assessment was well organised	38 / 39	37 / 41	0.241
I feel that I had enough time with the pre-operative assessment nurse	39 / 39	39 / 41	0.494
My pre-operative assessment has fully prepared me for my operation	38 / 39	36 / 41	0.493
I am fully satisfied with my pre-operative assessment	38 / 39	38 / 41	0.494
My pre-operative assessment was inconvenient	2 / 37	4 / 41	0.673

## Discussion

In this prospective, observational study, we have found that ePAQ-PO is associated with a 13 minute (50%) reduction in duration of nurse-led pre-operative assessment time in ASA physical class 1 and 2 patients. This is greater than the 5-minute reduction seen in a similar study which introduced a tablet-based application to their pre-operative assessment clinic [20]. When extrapolated to a clinic with 10 nurse practitioners working all day, with an average attendance time of approximately 60 min [21], this would allow 10 more patients to be seen per day, amounting to over 2,500 extra patient slots per year. Introducing a system which enables safe and efficient pre-operative assessment of low-risk patients, also allows greater time and resources to be focused on higher risk patients.

Data from a cohort of 1092 patients who completed ePAQ-PO at our institution, showed that the median (IQR [min-max]) time to complete ePAQ-PO was 13:50 (10:57–18:02 [5:29–78:26]) mins. It could be argued that the time saving from the shorter duration of nurse consultation is therefore borne by the patients. Whilst the potential cost-savings of an initiative such as ePAQ-PO are vital, it was important to show that this type of pre-operative assessment was acceptable to patients. It could be argued that the question ‘My pre-operative assessment has fully prepared me for my operation’ could only be fully answered in retrospect after the surgical procedure. However, the immediate patient perception of the ePAQ-PO pathway was an important quality metric. The study was also too small to evaluate other outcomes such as ‘on the day’ cancellations and perioperative complications secondary to inadequate preoperative assessment. Other studies have shown that the use of an electronic questionnaire can help in the quality as well as economy of pre-operative assessment. [22] The potential inconvenience for ePAQ patients of attending pre-operative assessment at short notice, for example, immediately following a surgical out-patient attendance is reflected in the scores that, although not reaching statistical significance, showed that more patients in the ePAQ-PO group felt that their overall assessment was too long. Improved information regarding this potential pathway prior to attending surgical outpatients might help address this issue, although any inconvenience needs to be balanced against the alternative of standard assessment involving a return to hospital at a later date. Audit data from our pre-assessment clinic has shown that the mean (SD) personal cost of a single pre-assessment clinic attendance is £12.00 (€13.70, US$15.30). Any initiatives to reduce this is likely to be welcomed by patients. A system of ‘one-stop’ ePAQ-PO pre-assessment enables urgent cases to be pre-assessed more rapidly meaning that earlier dates can be given for surgery and patients would need to take less time off work, which is likely to lead to other indirect cost savings such as less need for carers’ leave (for both patients and/or their friends and relatives).

Our study has several limitations and its non-randomised, observational design means that it is prone to selection bias. Although we purposely matched the two groups for age and all patients could read and understand English, the ePAQ-PO group may have inadvertently attracted and recruited more computer-literate patients. The potential confounding effects of patients being seen by different nurses was minimised by limiting the number of nurses to four in the standard group and two in the ePAQ-PO group. The effect of different surgical specialties on consultation time has been shown to be minimal [19] and so was unlikely to have influenced the result in a study that included only patients of ASA physical class 1 and 2. The examination time was extrapolated from recordings taken from only 50% of standard group consultations; the narrow IQR of 2 minutes with a median duration of examination of only 4 min means that this extrapolation is probably justified. Only a small number of nurses were included in this single site study, and none of the patients needed a physician assessment. These factors potentially limit the generalisability of the findings to other centres where physician assessment is standard or mandatory. Nearly 900,000 people in the UK are not proficient in the English language and this cohort of the population has poorer general health [23]. ePAQ-PO could conceivably remove the need for all patients to attend hospital; patients could complete ePAQ-PO at their convenience on-line and the responses could be followed up by a telephone consultation from the pre-assessment team, with any required observations and investigations performed in the community. It has been estimated that the implementation of remote pre-operative assessment in the UK could result in cost efficiency savings approaching £50 million [20].

The screening questions (Appendix 1) were used in the surgical outpatient clinics to ensure that minimal numbers of patients with ‘complicated’ co-morbidities were sent to the ePAQ-PO ‘on the day’ assessment service whilst implementing this new service and assessing its benefits. This questionnaire is no longer used as ePAQ-PO is now utilised in all ASA grade patients in our institution. Future work will look at the use of ePAQ-PO to facilitate pre-operative assessment in patients who are ASA physical class > 2. The benefit of routine questions with negative responses not needing to be repeated would mean that nurses could concentrate on areas of patients’ comorbidity that are important for pre-operative optimisation and could spend more time informing patients about what to expect on their admission to hospital. The use of ‘big data’ from a large database will allow studies to be powered to look not only at potential time savings but also at the relevance of any investigations ordered and a measure of ‘on the day’ cancellation rate or perioperative complications [24–26]. Future cohorts involving patient of higher ASA physical classes may be more likely to demonstrate a difference in these outcome measures.

## Conclusion

The use of ePAQ-PO is associated with a significantly shorter duration of nurse-led pre-operative assessment consultations in our centre, for patients who are ASA physical class 1 and 2. The introduction of this new service can potentially allow more low-risk patients to be pre-operatively assessed without a proportional increase in service costs and without any detrimental impact on patient satisfaction.

## Supporting information

S1 Appendix Screening questions used in surgical outpatients to determine eligibility for ePAQ-PO pathway. (DOCX)

S1 Data Study Data ePAQ. Excel spreadsheet with all raw (anonymised) data used in the study.
(XLSX)
