# Supplementary material for: A prospective observational study of the impact of an electronic questionnaire (ePAQ-PO) on the duration of nurse-led pre-operative assessment and patient satisfaction
Source: PLoS One. 2018 Oct 19;13(10):e0205439. doi: 10.1371/journal.pone.0205439 (PMC6195264; doi:10.1371/journal.pone.0205439)
Supplement: S1 Appendix — (DOCX) [file pone.0205439.s001.docx]

**Appendix 1. Screening questions used in surgical outpatients to determine eligibility for ePAQ-PO pathway.** Answering ‘yes’ to any of these questions means the patient is less likely to be ASA 1 or 2 and is therefore not invited to the ‘same day’ ePAQ-PO pathway.

- Taking more than three medications (excluding paracetamol and aspirin)?
- Breathlessness after climbing two flights of stairs?
- Ever seen a cardiologist (regardless of outcome)
- Ever had a myocardial infarction, angina or cerebrovascular accident
- Diagnosis of asthma or chronic obstructive pulmonary disease
- Diagnosis of Rheumatoid Arthritis
- History of pulmonary embolism or deep vein thrombosis
- Family history of bleeding disorders
- Neurological disorders
- Consume more than 30 units of alcohol per week
- Unable to use a computer mouse
- Unable to read or understand English to a suitable level.
